# Establishment of a New Cell Line of Canine Mammary Tumor CMT-1026

**DOI:** 10.3389/fvets.2021.744032

**Published:** 2021-10-12

**Authors:** Chen Mei, Liang Xin, Yang Liu, Jiabao Lin, Hong Xian, Xue Zhang, Wei Hu, Zhaofei Xia, Hongjun Wang, Yanli Lyu

**Affiliations:** ^1^Department of Clinical Veterinary Medicine, College of Veterinary Medicine, China Agricultural University, Beijing, China; ^2^Institute of Animal Husbandry and Veterinary Medicine, Beijing Municipal Academy of Agriculture and Forestry, Beijing, China; ^3^College of Veterinary Medicine, Veterinary Teaching Hospital, China Agricultural University, Beijing, China

**Keywords:** canine mammary tumor, tumorigenicity, establishment cell line, luminal triple-negative cell line, characterization

## Abstract

Canine mammary tumors (CMTs) have histopathological, epidemiologic and clinical characteristics similar to those in humans and are known to be one of the best models for human breast cancer (HBC). This research aimed to describe a newly established canine cell line, CMT-1026. Tumor samples were collected from a female dog exhibiting clinical mammary neoplasm, and the adherent cells were cultured. Both the histology and immunohistochemistry (IHC) of tumor samples were estimated. Cell growth, ultrastructural, cytological and immunocytochemistry (ICC) features of CMT-1026 were examined. CMT-1026 cells were inoculated into 10 female BALB/c nude mice to evaluate oncogenicity and metastatic ability. Hematoxylin-eosin (H.E.) staining of the tumors revealed an epithelial morphology. Electron microscopy was used to detect histological and cytological of smears, and ultrathin sections showed that CMT-1026 cells were polygonal and characterized by atypia and high mitotic index in the tumor, with prominent nucleoli and multinucleated cells. IHC characterization of CMT-1026 indicated ER-, PR-, HER-2, p63+, CK5/6+, and α-SMA+ epithelial cells. ICC characterization of CMT-1026 showed high expression of Claudin-1, Delta-catenin, SOX-2, and KI-67. At 2 weeks after inoculation of the CMT-1026 cells, phyma was found in 100% of the mice. The xenograft cancers showed conservation of the original H.E. features of the female dog cancer. In conclusion, CMT-1026 may be a model of canine mammary cancer that can be used in research on the pathogenesis of both CMT and HBC.

## Introduction

Triple-negative breast cancers (TNBCs) account for ~15% of all instances of mammary tumors without expression of estrogen receptor (ER), progesterone receptor (PR) and human epidermal growth factor receptor 2 (HER2). Therefore, TNBCs cannot be treated with hormonal modalities. Patients with non-TNBC usually show longer overall survival than those with TNBC (1–4).

CMT is one of the most common diseases in unneutered dogs (5). CMT has the second greatest tumor incidence rate after skin tumors. Approximately 50% of CMT cases are malignant (6), and CMT and HBC have similar epidemiological, histological, and clinical characteristics. CMT may serve as a good model for HBC study (7).

Chemotherapy and surgery remain the standard treatment regimen for TNBC. Compared with other breast cancer types, TNBC is more malignant and has poorer prognosis, but its response to chemotherapy is relatively good. Anthracyclines and taxanes are currently the most commonly used treatments in TNBC patients. The CAGLB 9344 study has shown that anthracycline sequential taxanes decrease the risk of recurrence and metastasis by ~1/3 in adjuvant therapy, as compared with only anthracyclines (8). In recent years, on the basis of traditional anthracycline and taxus drugs, researchers have continued to explore new research fields. New chemotherapeutic drugs, represented by platinum, gemcitabine, vinorelbine and capecitabine, have received increasing attention. Simultaneously, new prescriptions are being investigated to improve the curative effects in patients, and make progress in neoadjuvant, adjuvant and post-treatment (9–13). Many foundational studies have been reported on the internal heterogeneity of TNBC. For example, the commonly used PARP inhibitors in clinical settings, including olaparib and veliparib (14–18), PI3K Akt mTOR pathway inhibitors (19–25), nivolumab, pembrolizumab, durvalumab (26, 27), and avelumab (28–30), have achieved good results.

Cell lines can be used in many studies, thus enabling the examination of cell progression. Researchers have established more than six CMT cell lines obtained from primary tumors and metastatic tumors (31, 32). Unfortunately, in research on triple-negative CMT, the valid cell lines are restricted to CMT-7364 (33), which is not sufficient to study the mechanisms of different subtypes of triple negative mammary cancer.

Consequently, more than one CMT cell line must be established to explore pathogenesis and promote further research. The purpose of this research was to establish and identify the CMT-1026 cell line and to characterize its tumorigenicity.

## Materials and Methods

### Tumor Sample

CMT-1026 was established from a canine solid carcinoma procured after surgical removal of a 5-year-old teddy breed female dog. The neoplasm was clinically and histopathologically identified with a canine mammary tumor (CMT). The mammary glands showed firmness, thickening, warmth and pain. The original CMT was identified as a large solid carcinoma with three small granulitic tumors on the dermal surface. After surgical extraction, the tumor samples were rapidly soaked in phosphate-buffered saline (PBS) with penicillin-streptomycin solution (Gibco, USA). Cancer samples were processed for cell culture and histopathological confirmation.

### Establishment of the Tissue Culture Cell Line CMT-1026

Neoplasm tissues were ground and washed three times with PBS (Gibco, USA). Cancer samples were disaggregated with collagenase type II (Sigma-Aldrich, V900892) on a rocker under 5% carbon dioxide for 4 h. The disaggregated tissue suspension was placed in a 50 mL centrifuge tube and centrifuged at 1,000 rpm for 5 min, and the collected cells at the bottom of the tube were resuspended in Dulbecco's Modified Eagle Medium Nutrient Mixture Ham's F-12 (DMEM/F12) with 10% fetal bovine serum (FBS) (Gibco, USA) and 1% penicillin-streptomycin solution. The cells were added into 25 cm^2^ culture flasks (Corning, USA) and cultivated at 37°C in a humidified atmosphere containing 5% CO_2_. Cells were observed daily by microscopy.

When the cell cultures reached 90% confluence, the cells were incubated with 0.25% trypsin (HyClone, USA) containing EDTA. Secondary cultures were obtained from digested cells, and the cells were placed in new 25 cm^2^ flasks at a density of ~1 × 10^5^ cells/mL. The rest of the digested cells were mixed in DMEM/F12 with 20% FBS and 10% dimethyl sulfoxide (Sigma-Aldrich, USA) and stored at 4°C for 30 min, −20°C for 2 h and −80°C overnight. The cells were sampled and frozen every five passages. The next day, cells were placed in liquid nitrogen. CMT-1026 was considered to be established at passage 30.

### Cell Growth Assays

CMT-1026 was diluted to different concentrations to provide a normal curve diagram (34). Cells were seeded in triplicate in 96 well plates. After 24 h of cultivation, CMT-1026 cells were treated in triplicate with a Cell Counting Kit for 4 h (CCK-8, Beyotime, Shanghai, China) and then detected at 24 h intervals for 9 days. Then the different concentrations were determined with a 450 nm filter and a microplate reader (ELx808 Absorbance Reader, BioTek, USA). When cell growth was in the exponential phase, the CMT-1026 doubling time was calculated with GraphPad Prism 8 software (GraphPad Software, Inc. USA).

### Electron Microscopy

CMT-1026 cells were collected for 24 h and resuspended with 2.5% glutaraldehyde (Panreac, Germany) and 4% paraformaldehyde (Panreac, Germany) solution at 4°C for 16 h. Then cells were added into 1% OsO4 for 1 h, gently washed twice with PBS, treated with graded acetone solutions (30, 50, 70, 80, and 100%) and encapsulated in epoxy resin. Ultra-thin sections were prepared with a Reichert-Jung Ultracut E ultramicrotome (LEICAUC6i, Germany). Lead citrate and uranyl acetate were used to stain the cells, and a JEOL JEM 3000F transmission electron microscope (JEOL Ltd., Tokyo, Japan) was used to obtain photographs.

### Karyotyping

For karyotype analysis, colchicine (Gibco-Life Technology, USA) was added at 0.06 μg/mL for 6 h before the cell suspension was harvested. Then attached cells were dissociated, and cell suspensions were added into 0.075 M hypotonic KCl for 30 min. The cells were fixed with 1:3 acetic acid:methanol at 4°C overnight. Finally, the prepared cells were stained with Giemsa stain (Beijing Solarbio Science & Technology, China) for 10 min and examined under a microscope (Leica Microsystems GmbH, Germany).

### Immunofluorescence

An indirect immunofluorescence experiment was used to monitor the expression of Ki-67 (Thermo Fisher Scientific, 14-5698-82), SOX-2 (Thermo Fisher Scientific, MA1014), Delta-catenin (Thermo Fisher Scientific, MA5-16386) and Claudin-1 (Thermo Fisher Scientific, 374900) in the CMT-1026 cell line. Ki-67 and SOX-2 were diluted to 5 μg/mL; Delta catenin and Claudin-1 were diluted to 1 μg/mL. CMT-1026 cells were seeded in 24 well plates. When cell cultures reached 90% confluence, cells were treated with acetone:methanol (1:1) for 20 min. Primary antibodies were used to stain cells at 4°C for 16 h. Then CMT-1026 cells were washed with PBS and cultured with secondary antibody for 1 h. The secondary antibody was Alexa Fluor 488 goat anti-rabbit IgG (Abcam, ab150077) or FITC goat anti-mouse IgG (Abcam, ab6785), which was diluted to 1 μg/mL. Finally, cells were washed with PBS and incubated with DAPI (BD Transduction Laboratories, USA). A fluorescence microscope (CKX41, Olympus Corporation, Japan) was used to examine the cells.

### Immunohistochemistry (IHC)

For IHC assessment, CMT-1026 originating primary cancer and xenograft tumors were prepared in 3 μm samples. Cancer pellets were assessed for the following markers: cytokeratin 5/6 (CK5/6, Invitrogen, 53-9003-82), estrogen receptor (ER, Invitrogen, MA1-12692), progesterone receptor (PR, Invitrogen, PA5-82322), human epidermal receptor-2 (HER-2, Invitrogen, MA5-13105), α-smooth muscle actin (α-SMA, Invitrogen, 14-9760-82), and P63 (Invitrogen, 14-9760-82). All these makers were used at a concentration of 1 μg/mL. Deparaffinized tissues were placed in a PT module with EDTA buffer solution (pH 8.0) (Master Diagnostica, MAD-004072R/D) and subjected to heat-induced antigen retrieval at 95°C for 30 min, then cooled to 60°C. To quench endogenous peroxidase activity, the slides were covered with 3% hydrogen peroxide and incubated with 5% FBS for 1 h. The specimens were incubated with primary antibodies at 4°C for 16 h and with secondary antibody (ZSGB-BIO, Beijing, China) for 1 h at room temperature to allow for the identification of immunolabeled proteins. Subsequently, 3,3′-diaminobenzidine tetrahydrochloride (ZSGB-BIO, China) was used to visualize antibody binding. The samples were washed in distilled water three times, then counterstained with hematoxylin, dehydrated, cleared and mounted. The primary antibody was substituted with PBS for negative control samples. The primary CMT was considered positive for CK5/6, ER, PR, HER-2, SMA, and p63 when more than 10% of the tumor cells were positive (35). ImageJ (National Cancer Institute, USA) was used to quantify the protein in the IHC images.

### Invasion Assays

To evaluate the metastatic ability of CMT-1026 cells, Corning Transwell chambers (Corning Incorporated, USA) were coated with Matrigel (Becton, USA). Cells were digested before being seeded onto the filters at 1 × 10^5^ cells/well in 200 μL DMEM/F12 medium, and 600 μL DMEM/F12 medium containing 10% FBS was mixed in the lower chambers for 48 h. The cells that invaded to the bottom of the chamber were detected after Crystal Violet staining for 5 min and washing with PBS. Five randomly fields per well were chosen to count cells. The experiment was performed in triplicate.

### Scratch Assays

To study the migration of CMT-1026 cells, wound-healing assays were used. In this assay, a scratch is made on a confluent cells with a pipette tip (36, 37). Subsequently, migration was measured in ImageJ software, and the wound healing percentage was calculated.

### Tumorigenicity Assays

For murine xenotransplantation models, a cell suspension of 10^7^ in 0.2 mL PBS was inoculated into the left mammary fat pads of ten 5-week-old female Balb/SCID nude mice subcutaneously. The growth of tumors was monitored weekly. When goiters were observed, the width and length of the tumors were regularly monitored with calipers for 8 weeks. When mice exhibited dyspnea and weakness or there were no palpable tumors, they were sacrificed. Neoplasms and organs were collected in 4% paraformaldehyde (pH = 7.4) for histopathological detection. All procedures were conducted according to the Guide for the Care and Use of Laboratory Animals and conformed to the relevant EU Directive.

### Myoplasma Detection

Mycoplasma DNA was detected with a EZ-PCR Mycoplasma Detection Kit (Biological Industries, Israel) according to the manufacturer's instructions. The PCR products were then separated with 0.8% agarose gel electrophoresis and visualized under UV light (Gel DocTM XR, BIO-RAD, America).

### Statistical Analysis

The invaded cells were calculated as the mean ± standard deviation (mean ± SD). The results were analyzed in SPSS 20 software. When the *p* value was < 0.05, it was regarded as indicating a significant difference among groups.

## Results

### Establishing the CMT-1026 Cell Line From a Canine Mammary Tumor

**Figure 1:**
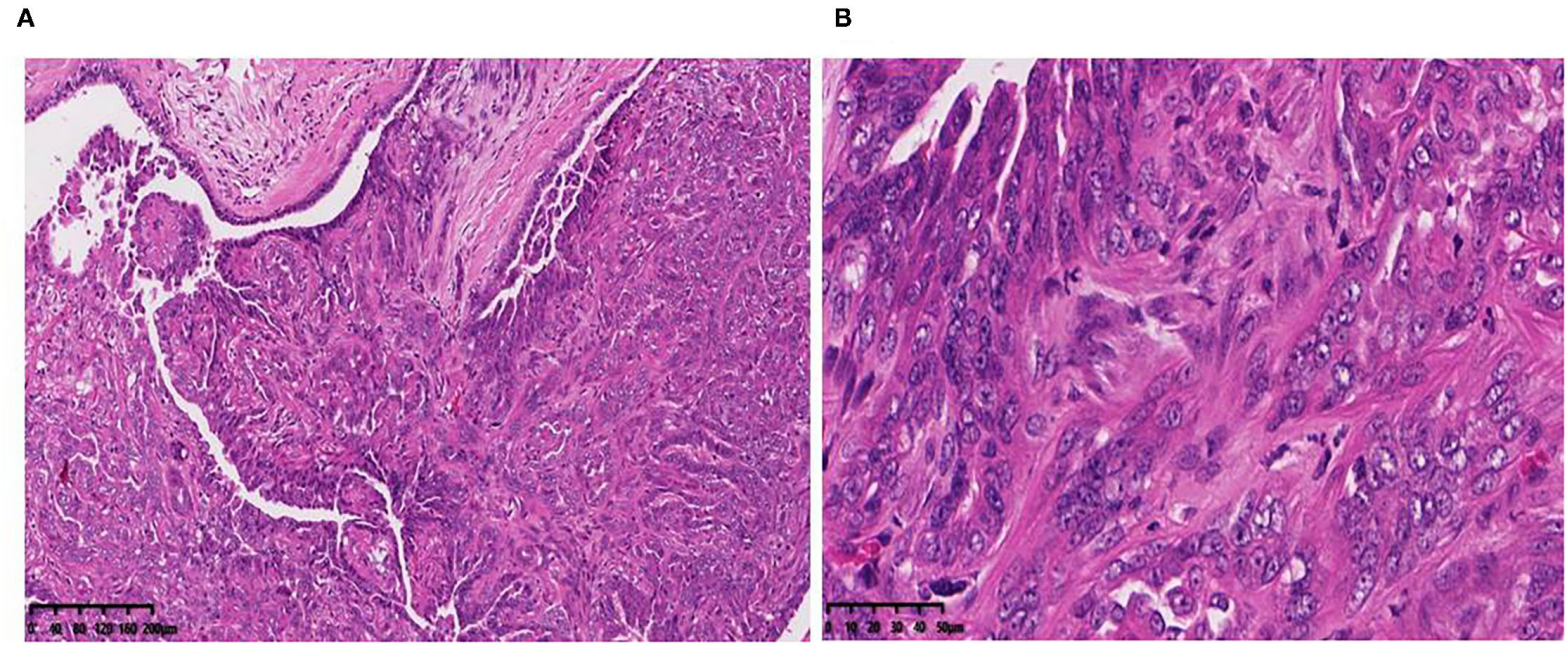
Primary CMT origin of the cell line CMT-1026. Tumor paraffin sections, stained with H.E. **(A)** (10×), **(B)** (40×). The tumor cells are hyperchromatic and show large nucleoli.

A canine mammary neoplasm was obtained from the China Agricultural University Veterinary Teaching Hospital. Histological observation of H.E.-stained paraffin sections from the tumor revealed substantial cell hyperplasia and elliptical, and round and polygonal cell shapes. Malignant multinucleated cells frequently had clearly enlarged nuclei. Anisocytosis, anisokaryosis, and elongated eccentric nuclei were observed (Figure 1). The CMT-1026 cells have been continuously sub-cultured for 54 generations.

### Immunohistochemical Characterization of the Canine Mammary Neoplasm

**Figure 2:**
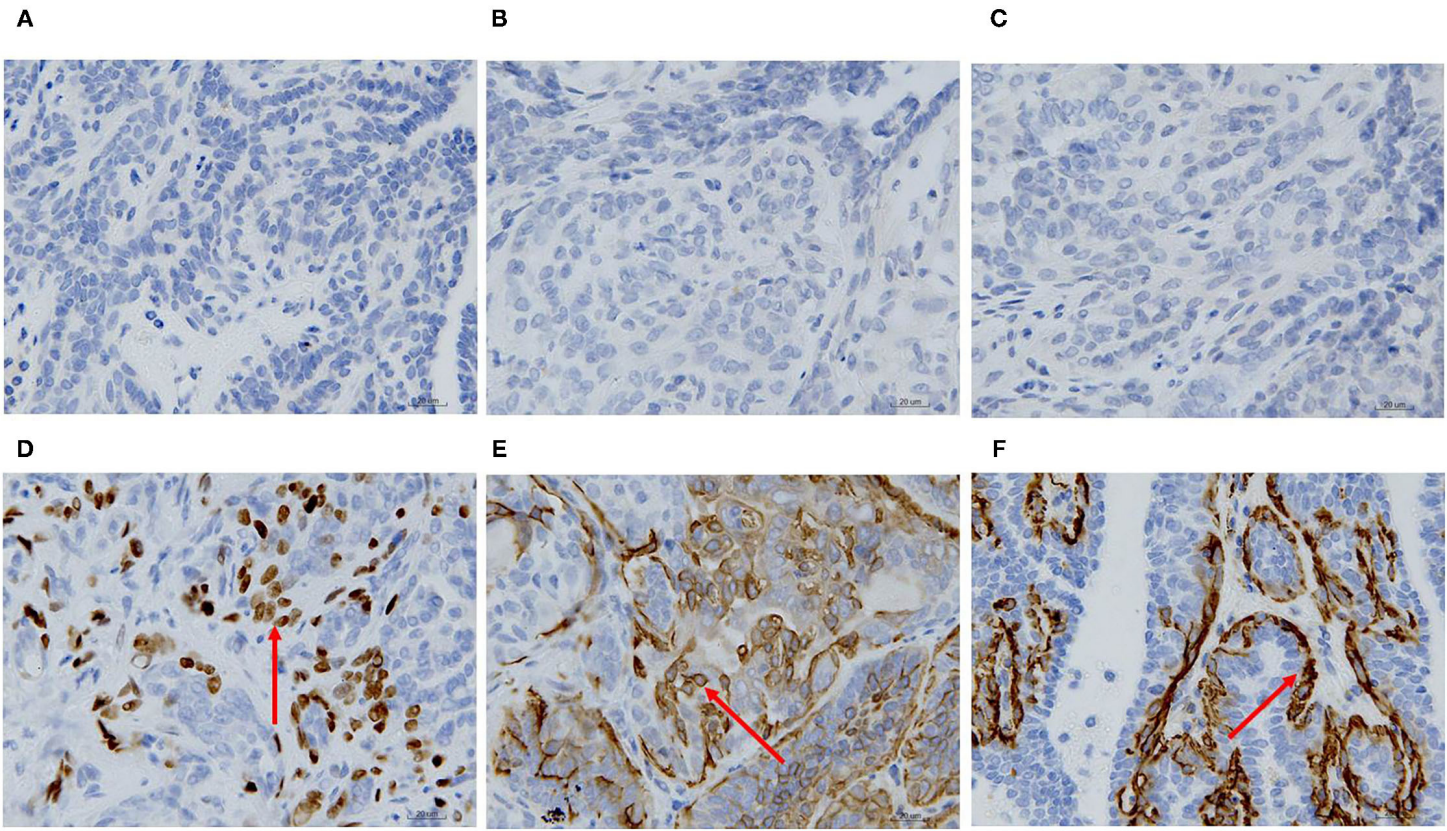
Immunohistochemical staining of the primary carcinoma sections (400X). The dark brown color was observed as positive staining. Tissues from the primary cancer were negative for ER **(A)**, PR **(B)** and HER-2 **(C)**, and were positive for P63 **(D)**, CK5/6 **(E)**, and α-SMA **(F)** localized in the cell membrane.

ER, PR, HER-2, P63, CK5/6, and α-SMA expression was detected through IHC in the primary CMT. The paraffin samples were negative for HER-2, ER and PR, but positive for CK5/6, P63 on the cell membrane and α-SMA in cytolymph (Figure 2). Image J was used to quantify the protein in the IHC images. P63 showed a positive expression rate of 12.89%, CK5/6 showed a positive expression rate of 29.45%, and α-SMA showed a positive expression rate of 23.17%. The neoplasm exhibited characteristics of a triple-negative canine mammary tumor.

### Purified CMT-1026 Cells and Observation by Electron Microscopy

**Figure 3:**
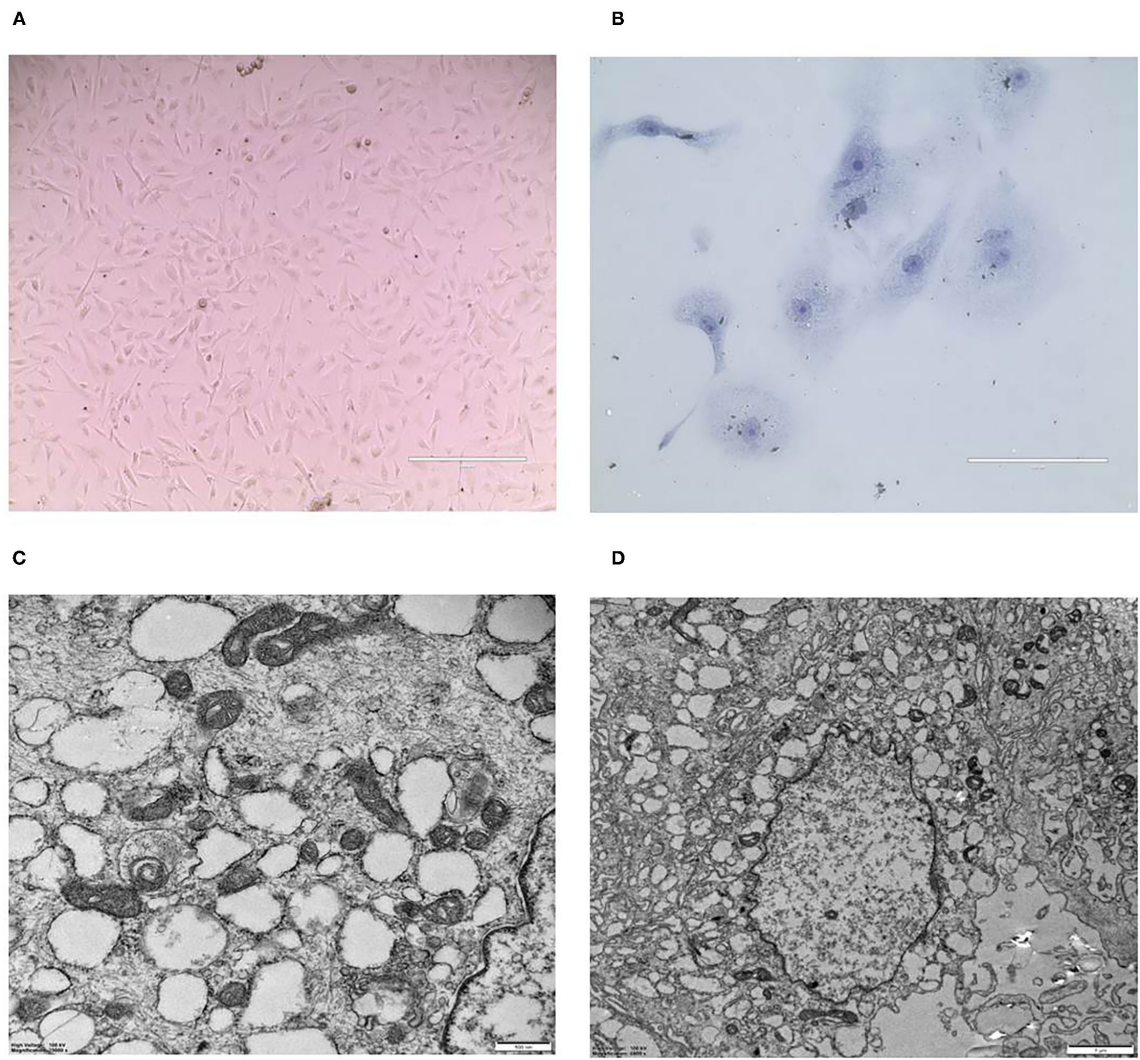
Morphology of CMT-1026 cells. **(A)** Purified CMT-1026 cells, detected with inverted microscopy (10×). **(B)** CMT-1026 pellet; Diff-Quik staining (40×). Malignant cancer cells with epithelial morphology, marked anisokaryosis, and multinucleated giant cells. **(C)** Ultrastructural experiments revealed that CMT-1026 contained multiple organelles, mitochondria, secretory vacuoles, and endoplasmic reticulum. **(D)** Degenerated CMT-1026 cells, showing shrunken nuclei, and abundant proteinaceous secretion.

**Figure 4:**
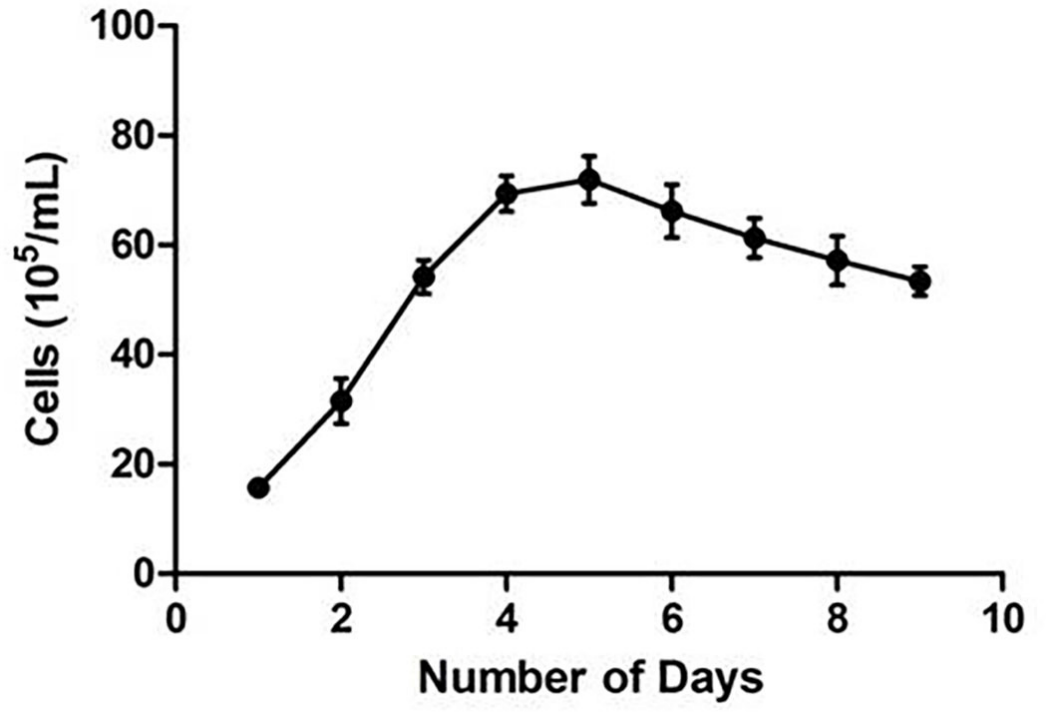
Growth curve of the CMT-1026 population. The doubling time was calculated to be 23.95 h.

CMT-1026 was purified and grown for more than 51 passages. The CMT-1026 cells were observed under light microscopy exhibited an epithelioid and spindle shape (Figure 3A). Diff-Quik staining showed malignant cancer cells with an epithelial morphology, marked anisokaryosis, and multinucleated giant cells (Figure 3B). Furthermore, ultrastructural studies revealed that CMT-1026 cells had large nuclei and apparent nucleoli. The cytoplasm contained multiple organelles, mitochondria, secretory vacuoles, and endoplasmic reticulum. Some vacuole structures were observed near the cell surface (Figure 3C). Degenerated tumor cells showed shrunken nuclei and abundant proteinaceous secretion (Figure 3D). The CMT-1026 doubling time was found to be 23.95 h (Figure 4).

### CMT-1026 Reveals Chromosomal Abnormalities

**Figure 5:**
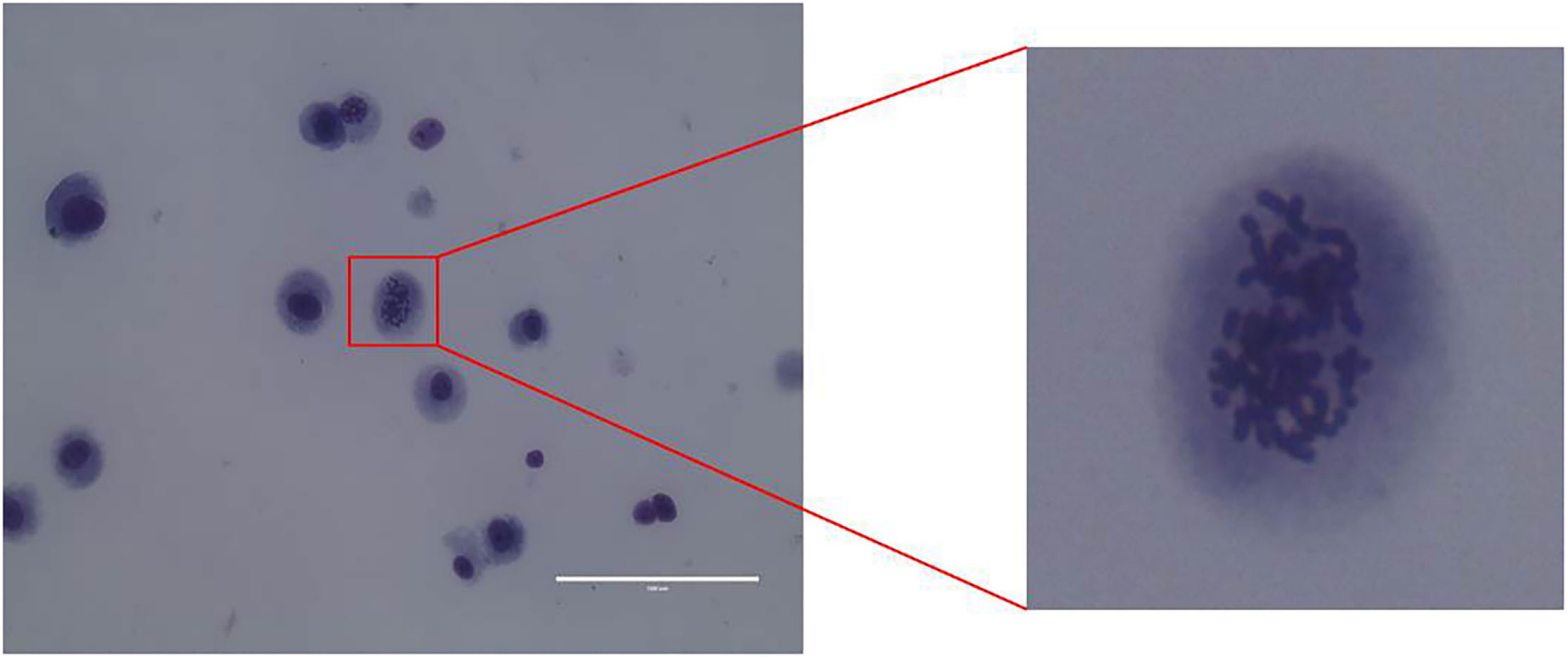
Karyotype analysis. The modal chromosome number in the cell line was 78.

Karyotype analysis was used to determine CMT-1026 cell chromosome numbers. Chromosome numbers were calculated on 80 near-diploid metaphase spreads. Most of the cells had 78 chromosomes, the chromosome number in canines (Figure 5).

### CMT-1026 Invasion and Migration Ability

**Figure 6:**
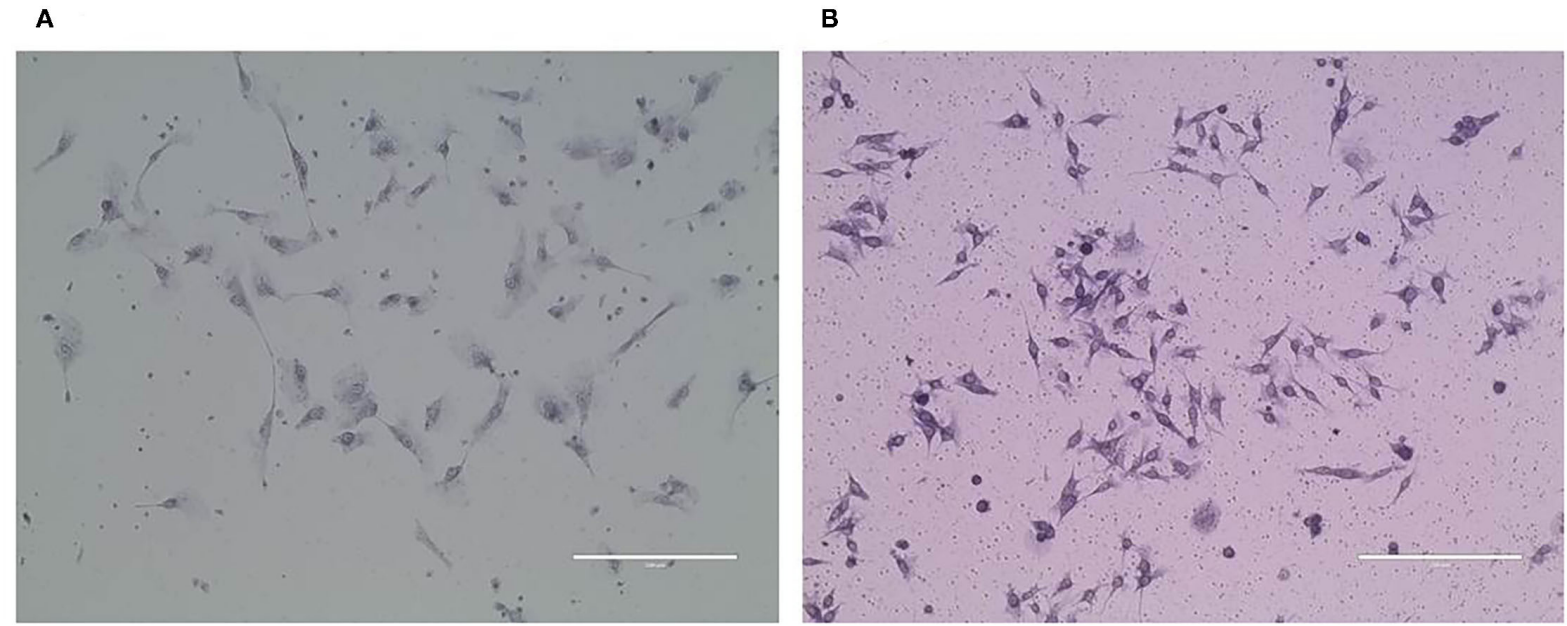
Invasion ability of CMT-1026 **(A)** compared with U27 **(B)** with Transwell assays.

**Figure 7:**
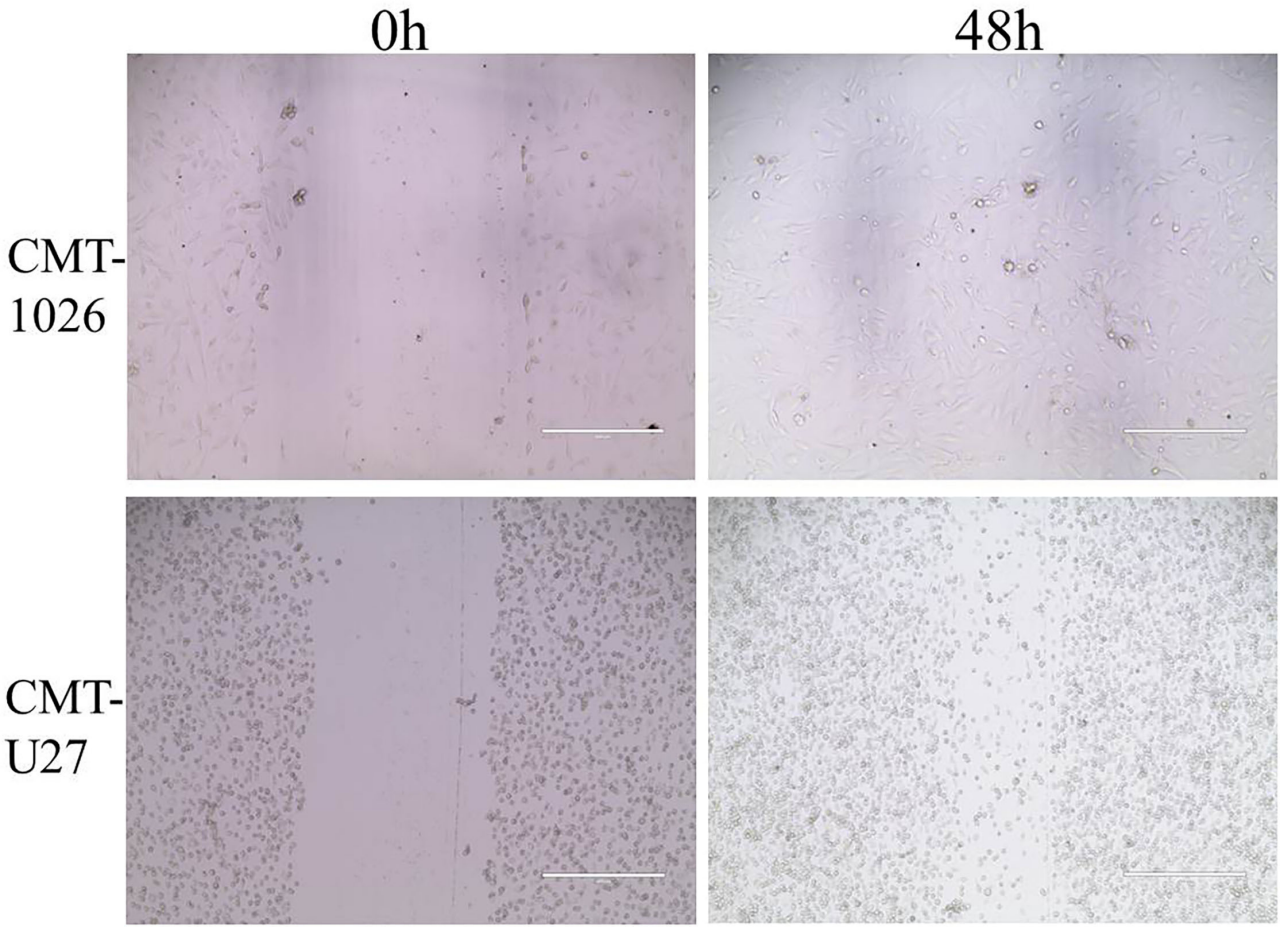
Characteristic pictures of wound healing assays at 0 and 48 h. At 48 h after the scratch, CMT-1026 reached a wound healing percentage of 80.4% ± 1.22 compared with 78.2% ± 1.43 for CMT-U27 (mean ± SD).

The CMT-1026 cells showed strong invasion ability through the basement membrane (63 ± 13) cells, whereas CMT-U27 cells showed an invasion ability of 172 ± 9 cells (Figures 6A,B). As predicted, 48 h after scratching, new growth CMT-1026 cells and CMT-U27 cells covered the scratched area. A wound healing percentage of 80.4% ± 1.22 in CMT-1026 was observed 48 h after the scratch. Notably, in comparison, the wound healing percentage for CMT-U27 was ~78.2% ± 1.43 after the same period of time (Figure 7).

### CMT-1026 Cells Have Strong Metastatic Ability

**Figure 8:**
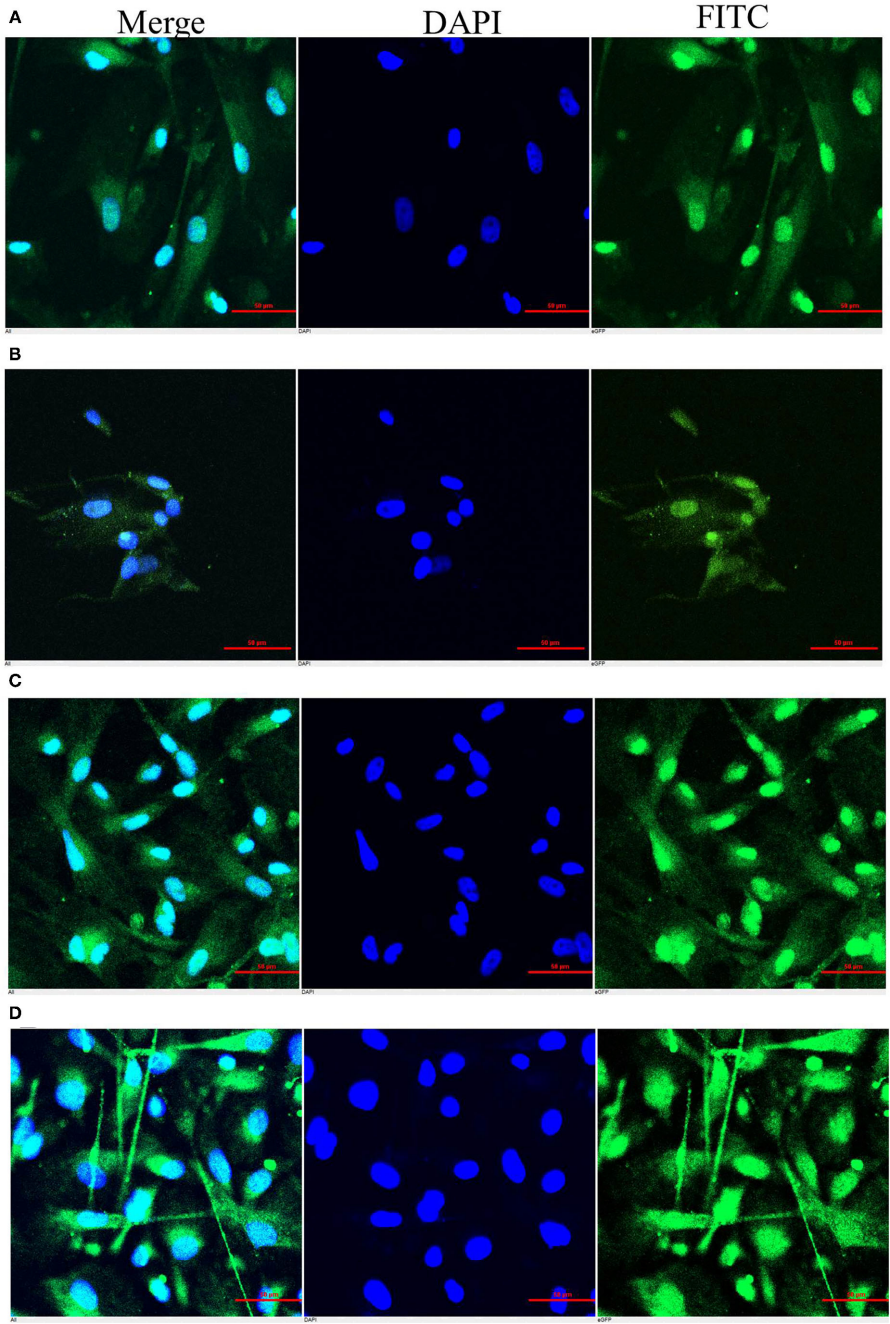
Immunocytochemistry of the CMT-1026 cell line. The cells showed positivity for Claudin-1 **(A)**, Delta-catenin **(B)**, Sox-2 **(C)**, and Ki-67 **(D)** in the cell membrane.

The expression levels of Ki-67, SOX-2, Delta-catenin, and Claudin-1 on CMT-1026 cells were observed by immunofluorescence. All samples were positive for these four antigens in the cytoplasm (Figure 8). The results indicated that the CMT-1026 cell line had a strong ability to metastasize.

### Tumorigenicity

**Figure 9:**
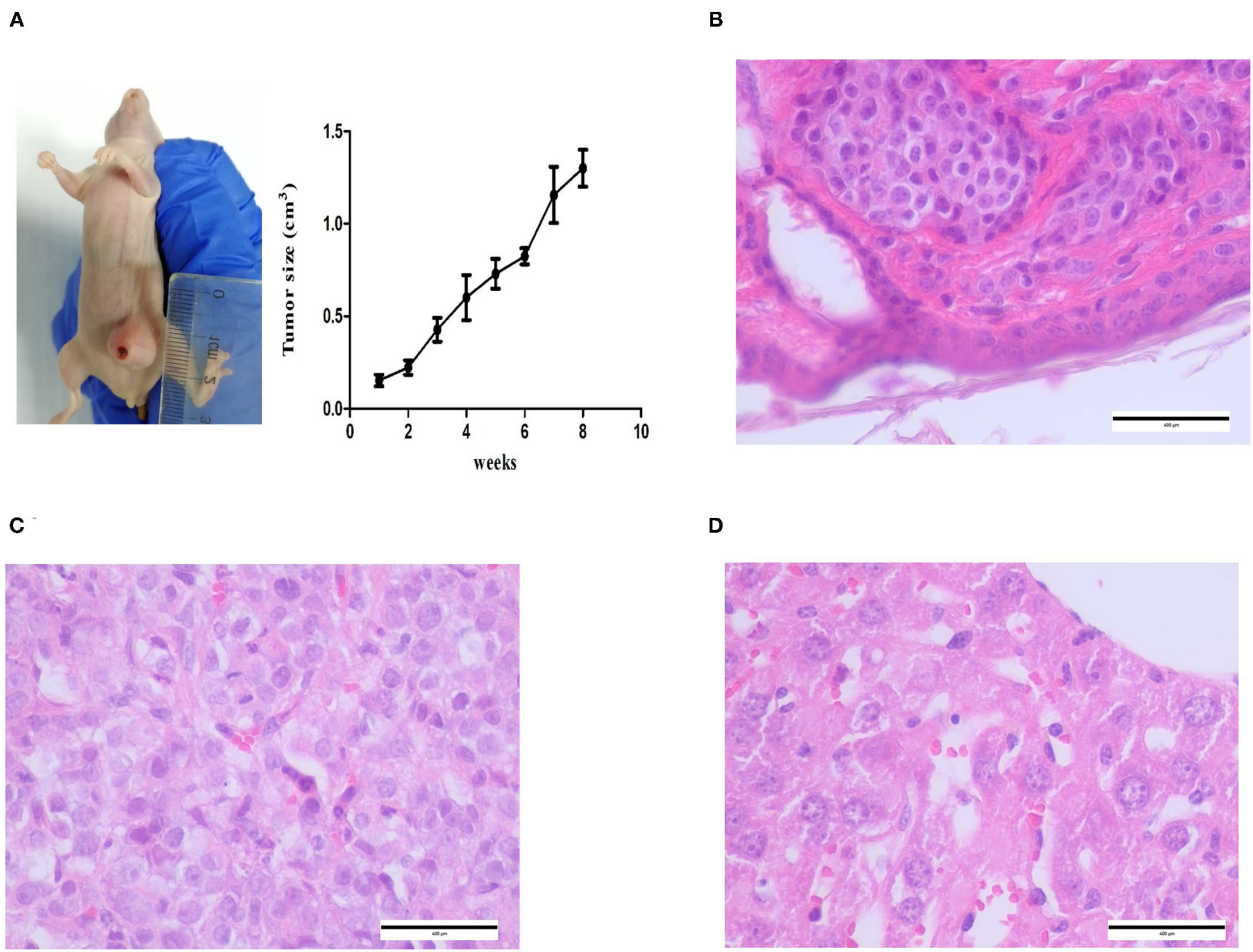
Mice inoculated with CMT-1026. **(A)** CMT-1026 mouse xenografts and tumor volume detection. **(B)** The subcutaneous dermis exhibited infiltrating cellular growth. **(C)** Glandular epithelial cells of lungs excised from xenografted mice. **(D)** Overall heteromorphism of liver cells was observed, along with increased nucleoli and necrosis.

CMT-1026 cells were injected into the abdomens of female BALB/c nude mice, and neoplasms grew 2 weeks after inoculation in 100% of the mice (*n* = 10, length: 0.5–0.8 cm, width: 0.2–0.3 cm). After 8 weeks of monitoring, the xenograft cancers were necrotic and ulcerated (length 0.8–1.3 cm; width 1.1–1.2 cm), and the mice were then euthanized (Figure 9A). Histological examination revealed infiltrating cellular growth in the subcutaneous dermis (Figure 9B). The lung tissue was invaded by glandular epithelial cells (Figure 9C). No clear tumor cell invasion was found in liver tissue, but the overall heteromorphism of liver cells was clear, the nucleoli had increased, and clear nucleoli and necrosis were observed (Figure 9D).

### Immunohistochemical Characterization of Xenograft Tumors

**Figure 10:**
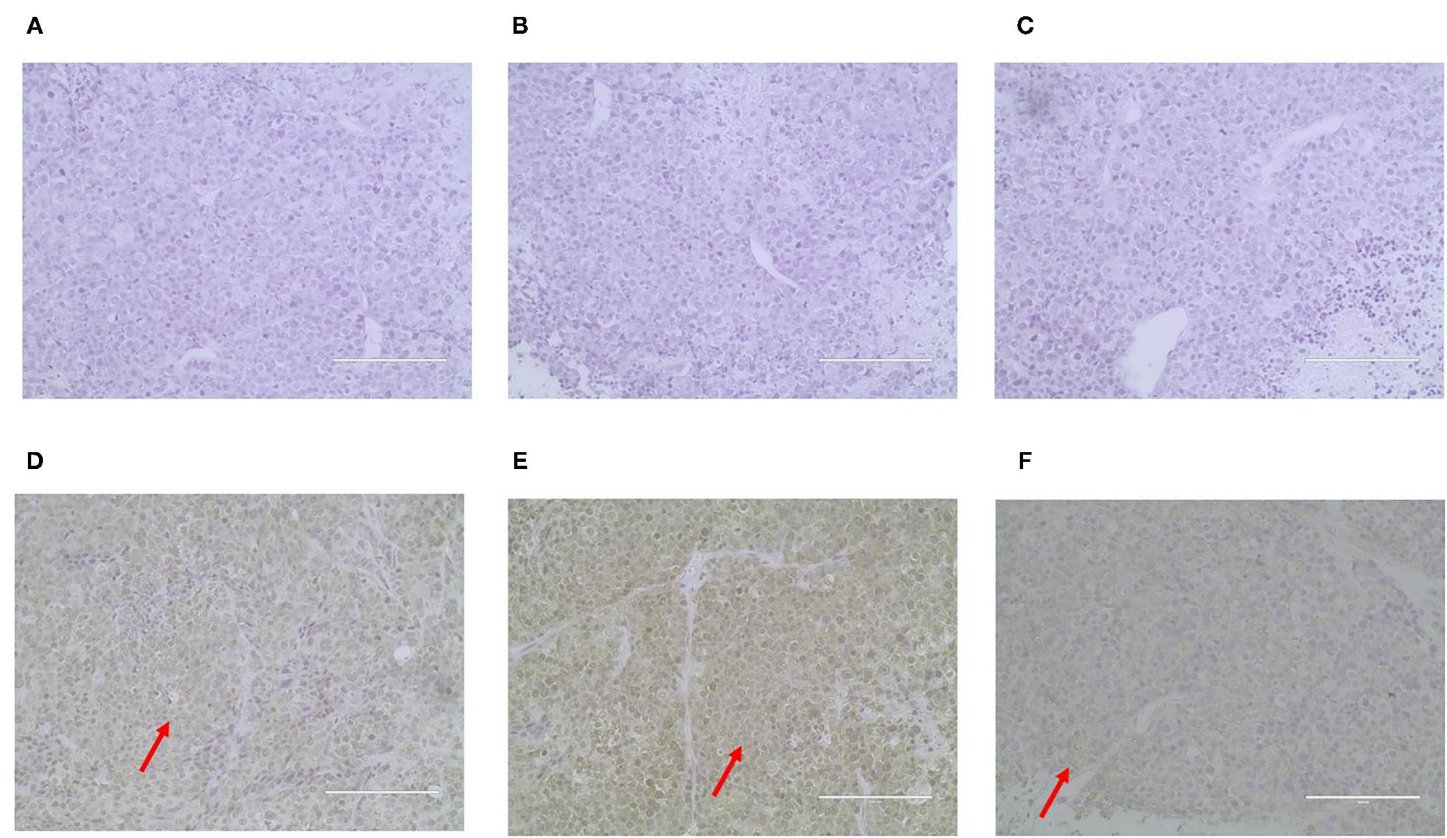
Immunohistochemical staining of the xenograft tumor sections (400X). Dark brown color was observed as positive staining. Tissues from xenograft tumors were negative for ER **(A)**, PR **(B)** and HER-2 **(C)**, and were positive for P63 **(D)**, CK5/6 **(E)**, and α-SMA **(F)** localized in the cell membrane.

ER, PR, HER-2, P63, CK5/6, and α-SMA expression was detected through IHC in the xenograft tumors. The results were consistent with the original tumor results. The paraffin samples were negative for HER-2, ER, and PR, but positive for CK5/6 and P63 on the cell membrane and for α-SMA in the cytolymph (Figure 10). Image J was used to quantify the protein in the xenograft tumors IHC images. P63 showed a positive expression rate of 38.94%, CK5/6 showed a positive expression rate of 50.01%, and α-SMA showed a positive expression rate of 52.35%. The xenograft tumors exhibited characteristics of triple-negative canine mammary tumors.

### Mycoplasma Detection

**Figure 11:**
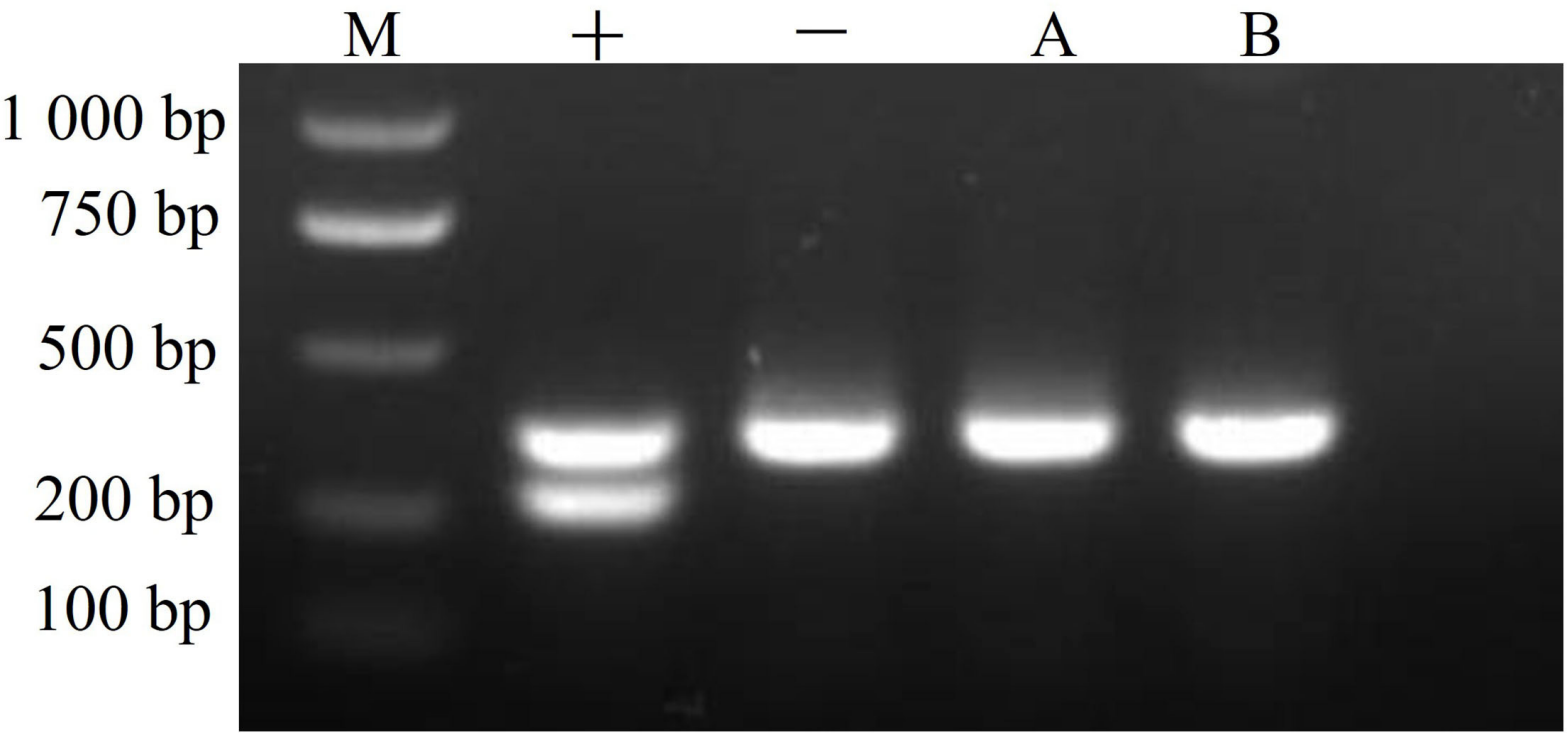
Mycoplasma detection. The CMT-1026 cell line exhibited no mycoplasma contamination. M, maker; +, positive control; –, negative control; A, culture media; B, CMT-1026 cells.

CMT-1026 cells were tested with a PCR-based method and found to be free of mycoplasma contamination (Figure 11).

## Discussion

The multiple treatments for dogs with CMT include chemotherapy followed by surgery. As in some canine mammary tumors, anti-hormonal targeted therapy is used in dogs positive for ER, PR, and HER-2 (38). The survival is shorter in TNBC cases that are particularly resistant to the anti-hormonal therapies (39). TNBCs account for 15% of mammary tumors and have a poorer therapeutic efficacy than that of hormone receptor positive disease (40–42). This type of CMT is also characterized by invasiveness and metastasis (43), and most cases are treated with chemotherapy (44). Chemotherapy is costly and has clear adverse effects; therefore, new treatments must urgently be developed in cell trials.

CMT cell lines are a favorable model for investigating molecular biology, heredity and tumor cures in many types of cancers containing TNBC cell lines (45). Developed CMT cell lines include CMT-1 to CMT-6 (46), CMT12, CMT27 (47), CMT-U27, CMT-U309, IPC-366 (48–50), and CMT-U229 (51). The canine TNBC cell line shares many pathological features with those of the illness in humans and thus offers an excellent animal model for human disease study (52–54). Nevertheless, only one triple negative canine mammary tumor cell line is available: CMT-7364, developed by Zhang et al. (33). Establishing more TNBC cell lines is important to provide a good model for immunotherapy research and mechanistic study.

Here, we established another TNBC cell line, CMT-1026. This cell line was then examined for its growth conditions, expression of different proteins and transplantation ability. Our IHC results revealed that CMT-1026 lacked expression of ER, PR, and HER-2 and therefore was another triple negative cell line, which may be a candidate for investigation of immunotherapy targets. IHC results indicated that CMT-1026 was a basal epithelial cell line, and its positive expression of cytokeratin 5/6 (for basal epithelial cells) (55), α-SMA (for myoepithelial cells) (56), and P63 (for myoepithelial cells) (57) indicated that CMT-1026 was of epithelial origin. CMT cells frequently show myoepithelial proliferation leading to preinvasive carcinoma (58, 59). CMT-1026 is also a well-established basal like subtype cell line (60). Comparative pre-clinical studies of CMT-1026 and IPC-366 cell lines may be possible in the future.

Delta-catenin expression in CMT-1026 has been demonstrated. Delta-catenin is a cell-cell adhesion protein in the Catenin family. Both mRNA and protein expression levels of Delta-catenin are higher in mammary tumor tissues than normal breast tissues. In addition, Delta-catenin expression is closely associated with histological grade and lymph node metastasis (61). Delta-catenin is an oncoprotein that is overexpressed in breast cancer, and its expression is indicative of poor prognosis (62).

Claudins (CLDNs) are the major transmembrane proteins forming tight junctions (63). CMT-1026 exhibited expression of Claudin-1. Loss of expression of tight junction proteins such as Claudin-1 might be assumed to lead to cellular metastasis and detachment, as commonly seen in breast carcinoma (64). Recent studies have suggested that Claudin-1 plays a major role in metastasis and invasion, and can be regarded as a diagnostic marker for mammary cancer (65).

CMT-1026 is also characterized by a high expression of Sox-2 and Ki-67. Sox-2 is a crucial transcription factor maintaining the multi-directional variation of stem cells. It has the ability to maintain cell self-renewal and proliferation, and it plays very important roles in embryonic development and the emergence and progress of malignant cancers (66). In recent years, studies have found that abnormal expression of Sox-2 is associated with the occurrence, variation, metastasis, and poor prognosis of malignant cancers (67). Ki-67 expression has been related to poor outcomes in malignant mammary cancers (68). The Ki-67 expression index in pathological reports is closely associated with the grade of variation, aggression, metastasis, and prognosis of many cancers. The higher the positivity rate, the faster the tumor proliferation, and the higher the degree of malignancy (69). Ki-67 and Sox-2 were found to exhibit high expression in the nuclei and cytoplasm of the tumor cells in this study. The nucleocytoplasmic staining was clear and stereoscopic. Because Ki-67 and Sox-2 high expression is conducive to cancer metastasis, the cells highly expressed Ki-67 and Sox-2 in this case, and the prognosis of this case appeared poor. CMT-1026 exhibited higher propagation ability than the original carcinoma, thus indicating a greater proliferative ability, possibly because the most malignant cells were selected.

CMT-1026 was compared with CMT-U27 and was found to have high propagation, as tested by Transwell and wound healing assays (70). CMT-1026 cells presented high growth and migration ability, and wound healing assays showed a 1.8% greater wound healing percentage than CMT-U27, possibly because CMT-1026 cells are larger than CMT-U27 cells. Ultrastructural characteristics (i.e., the secretory vesicles, multiple organelles, mitochondria, and endoplasmic reticulum) suggested that CMT-1026 is of epithelial origin.

The CMT-1026 cell line recapitulated the histological characteristics of the canine primary cancer. Then CMT-1026 was inoculated into BALB/c nude mice and caused carcinomas that displayed the histological characteristics of the original canine cancer. The original canine mammary carcinoma and CMT-1026 cell line showed similar histological malignancy features with marked anisokaryosis. CMT-1026 xenotransplantation in nude mice exhibited autonomous metastases in the lung and liver. Therefore, CMT-1026 can be considered a good model for research on the mechanism of metastasis in further studies.

## Conclusion

CMT-1026 may provide a valuable cell model for further research on malignancy, tumorigenicity and tumorigenic mechanisms. This newly established cell line has great research potential in advancing the progress of new therapeutic drugs.

## Data Availability Statement

The original contributions presented in the study are included in the article/Supplementary Material, further inquiries can be directed to the corresponding author/s.

## Ethics Statement

The animal study was reviewed and approved by Animal Care and Use Committee of the Institute (IACUC) under the approval of Institute of Animal Husbandry and Veterinary Medicine (Permit number: 2014-05).

## Author Contributions

CM, HW, and YLy conceptualized the paper. CM composed most of the manuscript. Figures were produced by CM, HW, LX, JL, HX, XZ, and WH. Critical revisions were made by CM, HW, LX, JL, HX, XZ, WH, ZX, and YLy. All authors agreed on the final manuscript.

## Funding

Project of the Beijing Academy of Agriculture and Forestry Science (Grant nKJCX20200211).

## Conflict of Interest

The authors declare that the research was conducted in the absence of any commercial or financial relationships that could be construed as a potential conflict of interest.

## Publisher's Note

All claims expressed in this article are solely those of the authors and do not necessarily represent those of their affiliated organizations, or those of the publisher, the editors and the reviewers. Any product that may be evaluated in this article, or claim that may be made by its manufacturer, is not guaranteed or endorsed by the publisher.
